# A Review of Lewis R. Rambo and Charles E. Farhadian’s *The Oxford Handbook of Religious Conversion*

**DOI:** 10.1007/s11089-016-0714-4

**Published:** 2016-06-16

**Authors:** Esra Ozyurek

**Affiliations:** London School of Economics, European Institute, Houghton Street, WC2 2AE London, England

**Keywords:** Religious conversion, German Muslims, Turkish Christians, Political and cultural conflict

## Abstract

This is a review of the *Oxford Handbook of Religious Conversion*. Drawing upon research on Germans converting to Islam and Turks converting to Christianity, converting is understood as complex fusion of individual choice and cultural/political conflict.

*The Oxford Handbook of Religious Conversion* (Rambo and Farhadian 2014) shows us that religious conversion is a profoundly complex experience and that all disciplines of the social sciences and humanities have something to say about it. It is a deeply individual journey yet a broadly social phenomenon. Scholars invited to contribute to the handbook repeatedly state that until now the psychological and individual dimensions of religious conversion have been emphasized and the social and especially the political aspects have been understudied. In my comments on this ambitious volume, I will try to attract further attention to the ways in which religious conversion can be a perfect place to look at the connections between the individual and the political and between the psychological and the cultural.

In my work on German converts to Islam (Ozyurek 2009a, 2015) and Turkish converts to Christianity (Ozyurek 2009b), I emphasize that voluntary religious conversion, especially to a minority religion, is one of the rare acts that grants individuals transformative power. Regardless of the intention of the convert, the act of conversion breaks established social, cultural, and political boundaries. In my fieldwork in both countries, I found that most of the time converts were oblivious of the power of their individual act and were often shocked at their being viewed as a social threat.

When Germans convert to Islam, they unmake what it means to be a German and also a Muslim. Contemporary German converts to Islam keep their German names and want to emphasize that one can be German and a Muslim. They give birth to and raise indigenous German Muslims who are neither converts nor immigrants. And, they make alliances with second- and third-generation immigrant Muslims and form German-speaking communities where they feel comfortable as both German and Muslim.

Similarly, when Turks convert to Christianity, they challenge the definition of Turkishness. In *Secularist Fears of a Converted Nation* (Ozyurek 2009b), I tried to explain why 3000 converts to Christianity in a nation of 70 million became the target of physical and political attack. Interestingly, it was not pious Muslims but rather secular nationalists who saw these converts as a major threat to the Turkish nation and the state. Reactions towards converts and the anti-missionary campaign in Turkey reveal that the foundation of Turkish nationalism has a religious component and assumes that only Muslim citizens can be loyal to the Turkish state.

In a comparative study of public anxiety towards converts in Germany and in Turkey, I argue that converts to minority religions mobilize new fears about the present and old anxieties about the past. “The convert alert triggers memories of the—by definition—unfinished job of national, religious, and cultural homogenization in nation-states and supra-national entities of the post-Cold War period. Essential to the establishment of both modern Turkey and modern Germany is the elimination of their most significant ethnic and religious minorities, namely Jews and Armenians, who were central elements of each society. Hence, in the current nationalist narratives dominant in both countries religious identity and especially that of the marked religious minority represents the past (Ozyurek 2009a). Hence, it is not a coincidence that in both countries, and in many others, converts to minority religions are feared and loathed as social threats to national integrity and security.

There is a budding interest in anthropology in the political meaning of conversion. In her work on a few hundred people who applied to the courts to change their names and religion to claim their Armenian descent that their ancestors had hidden in order to survive the Armenian Genocide one hundred years ago, Ceren Ozgul (2014) reveals the complicated implications of the seemingly simple desire of these individuals. Their desire to convert to Christianity on the basis that they are descendants of forcibly Islamized Armenians challenges the official Turkish denial of the Armenian Genocide. Similarly, Michal Kravel-Tovi (2012) and Don Seeman (2003) show the political consequences of conversion to Judaism in Israel. As Robert Hefner (1993) reminds us, religion has a crucial place in community, politics, and morality. Religious converts reveal the power of these categories as they unmake and make them again.

*The Oxford Handbook on Religious Conversion* is a wonderful testimony to the multiply complicated dimensions of the seemingly simple act of religious conversion. Many of the contributions highlight that the scholarly focus on religious conversion has focused too much on the decision-making process of the individual convert at the expense of the context in which the decision is made. Individual decision is not only a product of its context but more importantly is transformative of it—in ways that are not foreseeable by converts.
